# Structure-based dual affinity optimization of a SARS-CoV-1/2 cross-reactive single-domain antibody

**DOI:** 10.1371/journal.pone.0266250

**Published:** 2022-03-30

**Authors:** Traian Sulea, Jason Baardsnes, Matthew Stuible, Nazanin Rohani, Anh Tran, Marie Parat, Yuneivy Cepero Donates, Mélanie Duchesne, Pierre Plante, Guneet Kour, Yves Durocher

**Affiliations:** 1 Human Health Therapeutics Research Centre, National Research Council Canada, Montreal, Quebec, Canada; 2 Human Health Therapeutics Research Centre, National Research Council Canada, Ottawa, Ontario, Canada; Weizmann Institute of Science, ISRAEL

## Abstract

The SARS coronavirus 2 (SARS-CoV-2) spike (S) protein binding to the human ACE2 receptor is the molecular event that initiates viral entry into host cells and leads to infection and virus replication. There is a need for agents blocking viral entry into host cells that are cross-reactive with emerging virus variants. VHH-72 is an anti-SARS-CoV-1 single-domain antibody that also exhibits cross-specificity with SARS-CoV-2 but with decreased binding affinity. Here we applied a structure-based approach to affinity-mature VHH-72 for the SARS-CoV-2 spike protein while retaining the original affinity for SARS-CoV-1. This was achieved by employing the computational platform ADAPT in a constrained dual-affinity optimization mode as a means of broadening specificity. Select mutants designed by ADAPT were formatted as fusions with a human IgG1-Fc fragment. These mutants demonstrated improved binding to the SARS-CoV-2 spike protein due to decreased dissociation rates. Functional testing for virus neutralization revealed improvements relative to the parental VHH72-Fc up to 10-fold using a SARS-CoV-2 pseudotyped lentivirus and 20-fold against the SARS-CoV-2 authentic live virus (Wuhan variant). Binding and neutralization improvements were maintained for some other SARS-CoV-2 variants currently in circulation. These improved VHH-72 mutants are predicted to establish novel interactions with the S antigen. They will be useful, alone or as fusions with other functional modules, in the global quest for treatments of COVID-19 infections.

## Introduction

The emergence at the end of 2019 of the severe acute respiratory syndrome coronavirus 2 (SARS-CoV-2, also termed CoV-2 in this paper), which causes the coronavirus disease COVID-19 [1], has led to a worldwide pandemic that two years later counts over 300 million confirmed cases and over 5.5 million associated deaths. At the time of this writing, more than one thousand monoclonal antibodies against SARS-CoV-2 have been reported in the literature [2], with over 200 antibodies in clinical evaluation and a few antibodies already approved for treatment [3, 4]. To maximize neutralization capacity, most of the antibodies in development are directed toward the receptor binding domain (RBD) of the SARS-CoV-2 spike (S) protein, in order to disrupt interaction between the viral S protein and its host cell receptor ACE2 [5]. These recombinant antibodies block viral entry by binding various epitopes on the RBD in a manner that fundamentally differs from the binding of the S glycoprotein to ACE2 and are therefore susceptible to viral mutational escape in current and emerging variants of SARS-CoV-2. These variants have already been shown to affect antibody neutralization potencies [6–8].

With a view to reduce the risk of mutational escape, there is a need to explore approaches for targeting more conserved epitopes. One way to accomplish this is to identify antibodies that cross-react with different sarbecoviruses that are phylogenetically more distant than their corresponding variants. In the case of SARS-CoV-2 variants, an opportunity is presented by SARS-CoV-1 (also termed CoV-1 in this paper), a zoonotic betacoronavirus that caused the 2004 SARS outbreak [9], and which also engages ACE2 as a cellular receptor. A maximum likelihood phylogeny analysis of the sarbecovirus S RBDs indicated that SARS-CoV-1 clade is distinct from the SARS-CoV-2 clade [10]. Two anti-RBD SARS-CoV-1 antibodies capable of binding the S RBD of SARS-CoV-2, albeit with lower affinity, have been discovered and characterized structurally and functionally. These include the single-domain antibody VHH-72 [11] and the monoclonal IgG antibody CR3022 [12, 13]; however only VHH-72 was found to be capable of neutralizing SARS-CoV-2. Structural analysis of VHH-72 bound to SARS-CoV-1 S RBD suggested that VHH-72 is able to cross-react with SARS-CoV-1 and SARS-CoV-2 by binding to a relatively conserved epitope on the S RBD. This epitope does not, however, appear to overlap with the ACE2 binding site on the S RBD. Rather, ACE2 would clash with the framework region of VHH-72, as opposed to classical receptor blocking in which the complementarity determining region (CDR) would occupy the ACE2 binding interface. VHH-72 binds to the SARS-CoV-1 S RBD through an H-bonding network involving CDR loops 2 and 3, in which backbone groups participate extensively [11]. Although this network is likely to be conserved in the case of SARS-CoV-2 S RBD binding, VHH-72 exhibited faster dissociation kinetics and reduced affinity in this case [11].

Here, using 3D-structural information, we optimized VHH-72 by applying ADAPT (Assisted Design of Antibody and Protein Therapeutics), a platform that interleaves structure-based computational predictions with experimental testing in order to optimize the binding affinity of a biologic for its target [14]. New in this work is the dual-affinity optimization, a.k.a. selectivity optimization, between antibody binding to multiple targets. Hence, we aimed to optimize the weak binding of VHH-72 to SARS-CoV-2 S protein while retaining or even improving the strong binding to SARS-CoV-1 S protein. Past applications of ADAPT affinity maturation have led to 10–100-fold binding affinity improvements for several antibody Fab and VH fragments [14, 15]. More recently, ADAPT was also applied for optimizing antibody binding selectivity towards the acidic environment of solid tumors relative to the physiological pH around normal cells [16]. ADAPT achieves these levels of affinity and selectivity optimization by significant focusing of the vast mutation space available. Depending on the size of the system, only a few dozen protein variants typically need to be produced, purified and tested. Lead designed mutants of VHH-72, formatted as Fc fusions, were characterized for *in vitro* neutralization of virus variants and *in vivo* efficacy in a hamster model of COVID-19 infection.

## Materials and methods

### *In silico* affinity maturation

The 2.2-Å crystal structure of the VHH-72 sdAb bound to the RBD of the SARS-CoV-1 S protein was downloaded from the Protein Data Bank (PDB ID: 6WAQ) [11], and was used as template to building a homology model of the VHH-72 sdAb bound to the RBD of the SARS-CoV-2 S protein (Wuhan sequence). Both complexes were refined by constrained energy minimization using the AMBER force field [17, 18], with a distance-dependent dielectric and an infinite distance cut-off for non-bonded interactions. Non-hydrogen atoms were restrained at their crystallographic positions with harmonic force constants of 40 and 10 kcal/(mol^.^A^2^) for the backbone and side-chain atoms, respectively, except for the amino-acid residues mutated in the SARS-CoV-2 homology model that were allowed to move freely. Resulting structures of the two complexes were used as starting points for tandem affinity maturation of the VHH-72. The ADAPT platform was then used for affinity maturation by single-point scanning mutagenesis simulations carried out at several positions within the CDR loops of VHH-72, as described previously for other systems [14, 15]. Briefly, we used three protocols, SIE-SCWRL [19–21], FoldX [22, 23] and Rosetta [24, 25], for building the structures and evaluating the energies of single-point mutations to 17 other possible natural amino acids (Cys and Pro were excluded) at these positions of the parental sequence. A consensus approach over specific versions of these three protocols was applied for building and scoring the VHH-72 mutants. Scoring of binding affinity was mainly based on the average Z-score and also on the average rank score over the scores calculated with the three component energy functions, SIE [20, 21], FoldX-FOLDEF [22] and Rosetta-Interface [24]. Further technical and implementation details of this consensus approach and its component methods can be found in Sulea et al [26]. Prior to binding affinity predictions, the FoldX-FOLDEF energy function [22] was used to estimate the effect of substitutions on the internal stability of the V_H_H structure. Thus, mutations predicted to be destabilizing by introducing folding free energy changes larger than 2.71 kcal/mol (i.e., 100-fold increase of unfolding equilibrium constant) relative to the parental molecule were discarded from further evaluation. Selection of designed mutants for experimental testing took into account the consensus Z-scores obtained in both complexes undergoing optimization. Preferred criteria for selecting particular mutations required an improvement in binding to SARS-CoV-2 RBD with a consensus Z-score below –1 together with an improvement in binding to SARS-CoV-1 RBD with a consensus Z-score below 0.

### Protein expression and purification

The DNA sequences of parental and mutated VHH-72 variants fused to human IgG1 Fc carrying the D270A mutation, codon-optimized for CHO cells, were synthesized and cloned into the pTT5^®^ plasmid by GenScript (Piscataway, NJ). Productions were carried out by transient transfection of CHO^55E1^ cells as described previously [27], at scales between 25 mL and 500 mL. Briefly, cells were transfected at a viable cell density of ~6×10^6^/mL with 1.4 μg of total DNA and 10 μg PEI MAX (Polysciences, Inc., Warrington, PA) per mL. Transfected DNA consisted of a 8.5:1.0:0.5 ratio of VHH-72-Fc, Bcl-xL (anti-apoptotic) and GFP (transfection marker) expression vectors, respectively. After addition of DNA:PEI polyplexes, cell cultures were incubated for 24 h on an orbital shaking platform at an agitation rate of 120 rpm at 37°C in a humidified 5% CO_2_ atmosphere. Cultures were then fed with Feed 4 (Fujifilm Irvine Scientific, Santa Ana, CA) at 2.5% v/v and anti-clumping supplement (Fujifilm Irvine Scientific) at 1:500, and transferred to 32°C. Cultures were supplemented again with Feed 4 (5% v/v) at 5 days-post-transfection and then harvested at day 7. Cell density and viability were determined by direct counting of cell samples with a Cedex automated cell counting system (Roche Innovatis, Bielefeld, Germany) using the trypan blue dye exclusion method. Post-transfection cell densities remained between 0.5–2×10^7^ cells/mL with viability greater than 89%. Titers ranged between 351–528 mg/L among the VHH-72-Fc variants.

Cell cultures were harvested by centrifugation at 20 minutes at 4,000 rpm and supernatants were then filtered using 0.2 μm Stericup or Steriflip vacuum filtration units (MilliporeSigma, Burlington MA). Purifications from cell-culture supernatants were performed by protein-A affinity chromatography, using 1-, 5- or 10-mL MabSelect SuRe columns (Cytiva Life Sciences) depending on production scale. Columns were equilibrated in HyClone^TM^ Dulbecco’s phosphate-buffered saline (DPBS; Cytiva Life Sciences). Supernatants were loaded at a residence time ≥ 3 min. Columns were washed in DPBS and eluted with 100 mM citrate buffer pH 3.6. Neutralization was achieved by supplementation with 1M HEPES (10% [v/v] final). Fractions containing the VHH-72-Fc variants were pooled and the citrate buffer was exchanged against DPBS using Zeba Spin (ThermoFisher Scientific) or HiPrep 26/10 desalting columns (Cytiva Life Sciences) and sterilized by filtration through 0.2 μm filters. Ultra-high performance liquid chromatography-size exclusion chromatography (UPLC-SEC) was used to assess the purity of all eluates. Homodimer content after protein-A affinity purification and desalting was higher than 99% according to UPLC-SEC analysis. Variants used for *in vivo* studies were concentrated at approximately 1.3 mg/mL or 6.7 mg/mL by ultrafiltration using Vivaspin Turbo centrifugal concentrator (Sartorius) with a membrane molecular weight cut-off of 10 kDa at room temperature following the manufacturer’s instructions. During the process, the protein concentration was monitored by measurement of absorbance at 280 nm and the calculated specific extinction coefficient of each variant. All the formulated samples for *in vivo* studies have an estimated homodimer percentage higher than 98.5% and endotoxin levels below 0.1 EU/mg as measured using the Endosafe system and FDA-licensed cartridges (Charles River Laboratories).

### Binding affinity measurements

Purified VHH-72-Fc variants were analyzed for binding to the S RBD domains of SARS-CoV-1, SARS-CoV-2 Wuhan, SARS-CoV-2 B.1.351 and SARS-CoV-2 B.1.617.2 using a Biacore T200 surface plasmon resonance (SPR) instrument (Cytiva, Marlborough, MA). Production and purification of these RBDs were carried out as described elsewhere [28]. The S RBD of SARS-CoV-2 B.1.1.529 was produced and purified following the same protocols. Samples were assayed at 25°C using PBS containing 0.05% Tween 20 (Teknova, Hollister, CA) with added 3.4 mM ethylenediaminetetraacetic acid (EDTA) and 0.05% Tween 20 as running buffer. S-RBD samples from SARS-CoV-2 (Wuhan, B.1.351 and B.1.617.2 variants) and SARS-CoV-1 were diluted to 10 μg/mL in 10 mM NaOAc immobilization buffer pH 4.5 (Cytiva, Marlborough, MA) and immobilized to approximately 350 RUs using the Immobilization Wizard for NHS/EDC amine coupling within the BiaControl software. The spike-RBD interactions were assessed using single cycle kinetics analysis for each variant with three concentrations using a 10-fold dilution from the top concentration of 100 nM. The VHH-72-Fc samples were injected at 50 μL/min with a contact time of 60 s and a 600-s dissociation. Sensorgrams were double referenced to the mock-activated blank sensor surface and analyzed for kinetic determination using a 1:1 binding model in BiaEvaluation software v3.1 (GE Healthcare). All VHH-72-Fc variants were run in triplicate. Due to avidity effects from the bivalent nature of the VHH-72-Fc variants, only dissociation rates, *k*_d_ (s^-1^), are reported.

### Thermal stability measurements

Differential scanning calorimetry (DSC) was used to determine the thermal transition midpoints (*T*_m_) of the parental and mutant VHH-72-Fc variants as previously performed [29]. DSC was carried out in a VP-Capillary DSC system instrument (Malvern Instruments Ltd, Malvern, UK). Samples were diluted in DPBS buffer to a final concentration of 0.4 mg/mL. DPBS blank and sample scans were carried out by increasing the temperature from 20°C to 100°C at a rate of 60°C/h, with feedback mode/gain set at “low”, filtering period of 8 s, pre-scan time of 3 min, and under 70 psi of nitrogen pressure. All data were analyzed with Origin 7.0 software (OriginLab Corporation, Northampton, MA). Thermograms were corrected by subtraction of corresponding DPBS blank scans and normalized to the protein molar concentration. The *T*_m_ values were determined using automated data processing with the rectangular peak finder algorithm for *T*_m_.

### Pseudovirus neutralization assay

Pseudotyped SARS-CoV2 spike lentiviral particles were produced using either the pHDM-SARS-CoV-2 Wuhan-Hu-1 expressing the SARS-CoV-2 Wuhan-Hu-1 spike protein (GenBank # NC_045512) or pcDNA3.3-SARS2-B.1.617.2 expressing the SARS-CoV-2 B.1.617.2 (Delta variant) spike protein, a gift from David Nemazee (Addgene plasmid # 172320) under a CMV promoter and packaged into lentiviral vectors obtained through BEI Resources, NIAID, NIH: SARS-Related Coronavirus 2, Wuhan-Hu-1 Spike-Pseudotyped Lentiviral Kit, NR-52948 and according to the protocols and reagents described by the Bloom lab [30], with the following modifications: (1) HEK293SF-3F6 cells [31] were used for large-scale production of lentiviral particles in 300 mL; (2) post-transfection HEK293SF-3F6 cells were incubated at 33°C for improved yield; (3) 72 h post-transfection lentiviral particles were harvested and subjected to concentration by sucrose cushion centrifugation. Briefly, the supernatant was placed on 20% sucrose cushion and spun for 3 h at 37,000×*g* at 4°C. The pellet containing the concentrated pseudotyped VLP was then resuspended in DMEM with 10% FBS and aliquoted. Titration was performed using HEK293T cells overexpressing human ACE2 and TMPRSS2, obtained from BEI Resources repository of ATCC and the NIH (NR-55293). Pseudovirus neutralization assay was performed according to the previously described protocol [30] and was adapted for 384-well plate. Briefly, 3-fold serial dilutions of the VHH-72-Fc samples were incubated with diluted virus at a 1:1 ratio for 1 h at 37°C before addition to HEK293-ACE2/TMPRSS2 cells. Infectivity was then measured by luminescence readout per well. Bright-Glo luciferase reagent (Promega, E2620) was added to wells for 2 min before reading with a PerkinElmer Envision instrument. 50% inhibitory concentration (IC50) were calculated with nonlinear regression (log[inhibitor] versus normalized response–variable slope) using GraphPad Prism 8 (GraphPad Software Inc.).

### SARS-CoV-2 microneutralization assay

SARS-CoV-2 isolate Canada/ON/VIDO-01/2020 was obtained from the National Microbiology Laboratory (Winnipeg, MB, Canada) and propagated on Vero E6 cells and quantified on Vero cells. Whole viral genome sequencing was carried out to confirm exact genetic identity to original isolate. Passage 3 virus stocks were used. Neutralization activity was determined with the microneutralization assay. In brief, 1:5 serial dilutions of 15 μg of each antibody was carried out in DMEM, high glucose media supplemented with 1 mM sodium pyruvate, 1 mM non-essential amino acids, 100 U/mL penicillin-streptomycin, and 1% heat-inactivated fetal bovine serum. SARS-CoV-2 was added at 125 plaque forming units (PFU) in 1:1 ratio to each antibody dilution and incubated at 37°C for 1 h. After incubation, Vero E6 cells seeded in 96-well plates were infected with virus/antibody mix and incubated at 37°C in humidified/5% CO_2_ incubator for 72 h post-infection (hpi). Cells were then fixed in 10% formaldehyde overnight and virus infection was detected with mouse anti-SARS-CoV-2 nucleocapsid antibody (R&D Systems, clone #1035111) and counterstained with rabbit anti-mouse IgG-HRP (Rockland Inc.). Colorimetric development was obtained with *o*-phenylenediamine dihydrochloride peroxidate substrate (Sigma-Aldrich) and detected on Biotek Synergy H1 plate reader at 490 nm. IC50 was determined from non-linear regression using GraphPad Prism 9 software.

### Hamster challenge

Male Syrian golden hamsters (81–90 g) were obtained from Charles River Laboratories (Saint-Constant, QC, Canada). Animals were maintained at the animal facility of the National Research Council Canada (NRC) in accordance with the guidelines of the Canadian Council on Animal Care. All procedures performed on animals in this study were in accordance with regulations and guidelines reviewed and approved in animal use protocol 2020.06 by the NRC Human Health Therapeutics Animal Care Committee. Animal health was monitored daily and clinical scores determined according to NRC ethics board approved clinical scoring table. If animals reached what is considered a clinical score of C, with significant weight loss, lethargy and clear outward signs of significant illness, then the animals were monitored twice daily. Animals were humanely euthanized if weight loss equaled or surpasses ethics approved cut-off value or at the end of the experiment, whichever was reached first. Animals were euthanized by exposure to CO_2_. All procedures anticipated to induce pain to animals were carried out under anesthesia by isoflurane inhalation or ketamine/xylazine injection. As this is a therapeutic study to determine efficacy of test article to protect animals after infection, no efforts were made to alleviate disease symptoms as this will affect study results. Best efforts were made to minimize suffering such as limiting duration of disease progression with predetermined cut-off values for clinical score and weight whereby humane euthanization was required. Male hamsters were challenged intranasally with 1 x 10^4^ PFU of SARS-CoV-2 isolate Canada/ON/VIDO-01/2020. Four hours post-challenge, animals were administered intraperitoneally 150 μl of antibody at the dose of 10 mg/kg. Daily body weights were determined. Animals were euthanized at 5 days post-challenge and viral load was determined for lung tissues by plaque assay. Plaque assay was performed as previously described [32].

## Results and discussion

### Identification of single mutants with improved binding to the spike protein

In the first round of ADAPT, 476 single-point mutations to all natural amino acids except Cys and Pro were computationally evaluated in the CDRs of VHH-72. The scanned region covered 28 positions (S30-E31 from CDR1, S52-K64 from CDR2, and G96-D100g from CDR3) that when substituted have the potential to alter the antigen-binding affinity.

**Fig 1:**
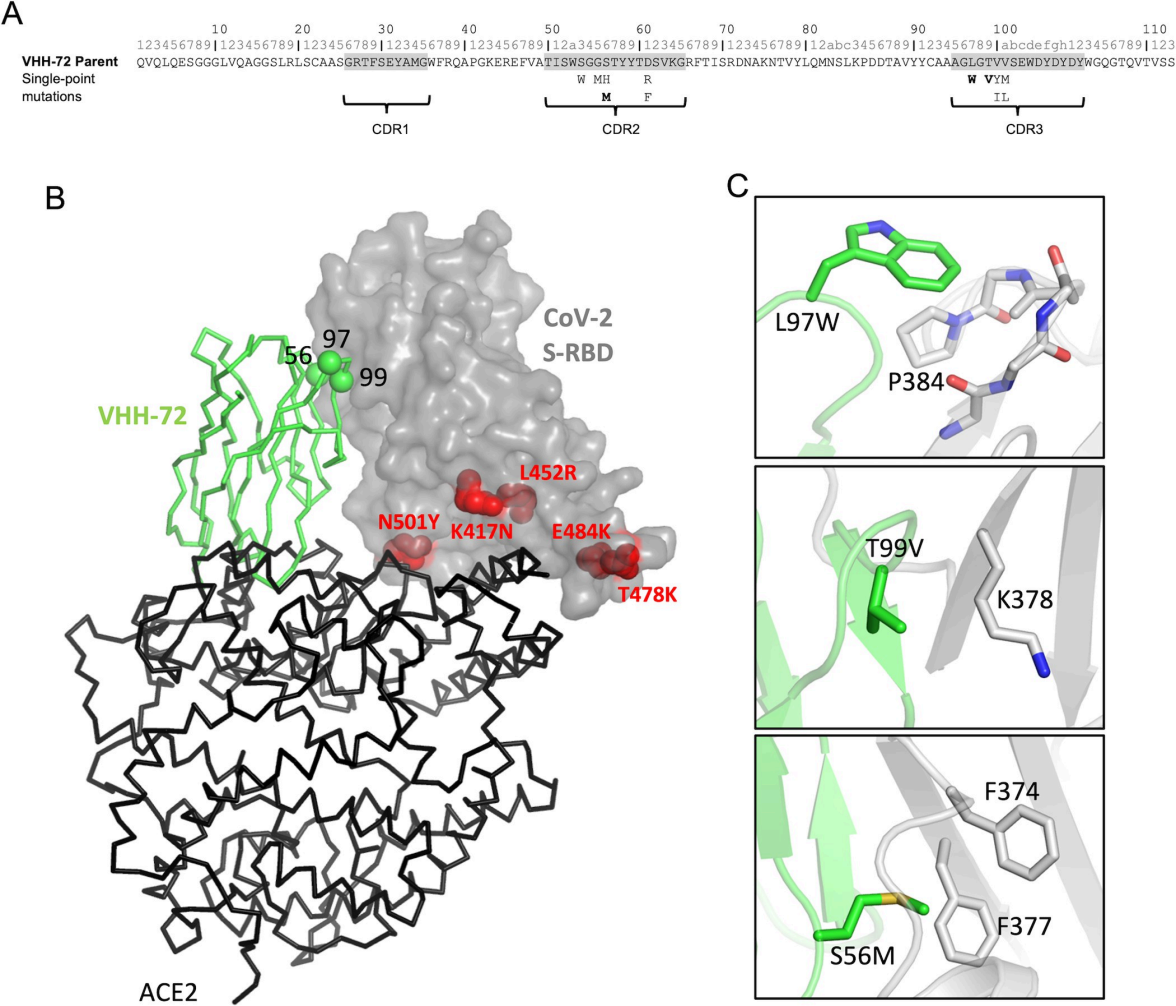
Structural details of designed VHH-72 mutations. (**A**) Sequence mapping of ADAPT-designed 13 single mutations tested experimentally, which are shown under the sequence of the parent VHH-72 (Kabat numbering shown on top). Mutant hits validated experimentally are bolded. (**B**) Structural context of the mutations at the positions corresponding to the 3 single-mutant hits (green Cα spheres labeled) of the VHH-72 (green Cα trace). Positions of the current SARS-CoV-2 mutations of concern from the B.1.351 (Beta) and B.1.617.2 (Delta) virus variants are rendered as red CPK models and labeled on the SARS-CoV-2 Wuhan spike-RBD (gray surface). The relative position of the ACE2 receptor ectodomain (black Cα trace) bound to the SARS-CoV-2 S RBD is taken form PDB entry 6M17. The steric clash between VHH-72 and ACE2 leading to their mutual exclusion when bound to S RBD is apparent from this superposition. (**C**) Detailed interactions at the three designed mutation sites. VHH-72 rendering is in green cartoon and C atoms, S RBD rendering is in gray cartoon and C atoms.

The selection of the most likely single-point mutants with improved antigen-binding affinities was primarily guided by the best ADAPT consensus Z-scores to both SARS-CoV-1 and SARS-CoV-2 S RBDs. The imposed score-based criteria were stricter towards improving binding affinity for CoV-2, for which the parental antibody had weaker binding than for CoV-1. Hence, single mutants were first selected to have consensus Z-score ≤ –1.0 for CoV-2 and ≤ 0 for CoV-1. This dual-score based selection retained 25 mutations at 9 positions in the CDR loops 2 and 3 (**Table 1**). A second selection step was then applied to retain some of the best scoring mutations at each position, in addition to reducing redundancy in the physico-chemical nature of the mutation for cases where multiple mutants were proposed at a given position. Visual inspection of structural models was applied to prune mutations with apparent poor steric and electrostatic complementarity, as in the case of mutations at position T57, and selection of the lower scoring mutation at position T99. The final list proposed for experimental testing in the first round included 13 single mutations at 8 positions, which were well balanced between CDR loops 2 (6 mutations at 4 positions) and 3 (7 mutations at 4 positions). This is a desired attribute in the set of single mutants that will increase the likelihood of additive contributions of mutation effects in the subsequent round of combining successful mutations. The list included single mutations S53W, G55M, S56H, S56M, D61R and D61F from CDR2 and L97W, T99V, V100Y, V100I, V100aM, V100aL and V100aI from CDR3. These mutations are mapped in **Fig 1A** on the amino-acid sequence of the parental VHH-72.

**Table 1:** ADAPT consensus Z-scores used in VHH-72 mutation selection.

CDR	Position	Mutation	Z-score		Selected	
			CoV-2	CoV-1	Round 1	Round 2
2	S53	W	-1.7	-1.6	+	
		Y	-1.0	-0.8		
	G55	M	-1.8	-1.6	+	
		W	-1.2	-1.2		
		L	-1.1	-1.1		
	S56	H	-3.2	-3.3	+	
		M	-2.4	-2.2	+	+
		Q	-1.3	-1.3		
		V	-1.1	-1.1		
		L	-1.1	-1.1		
		F	-1.0	-1.3		
	T57	H	-1.2	-1.1		
		W	-1.2	-1.1		
	D61	R	-1.7	-1.4	+	
		F	-1.6	-1.4	+	
3	L97	W	-2.3	-1.6	+	+
	T99	I	-1.2	-1.0		
		V	-1.0	-0.9	+	+
	V100	Y	-1.9	-1.5	+	
		F	-1.5	-1.6		
		I	-1.4	-0.8	+	
	V100a	M	-2.6	-2.3	+	
		L	-2.2	-2.3	+	
		I	-1.4	-1.4	+	
		H	-1.4	-1.2		

These 13 single-point mutants were produced recombinantly alongside the parental VHH-72 as fusions with a human IgG1-Fc fragment including a one-residue (alanine) linker, followed by the P226-P243 hinge region and the A244-G477 CH2 and CH3 domains (Kabat numbering was used throughout this paper). The C233 in the hinge region, normally used to link to the light chain of a conventional human IgG1 antibody, was mutated to serine in order to prevent formation of undesired covalent disulfide-bonded adducts. We also introduced the D270G mutation in the CH2 domain in order to attenuate immune receptor functions by reducing binding to human Fcγ receptors [33]. It is expected that this attenuation, rather than complete abrogation of immune receptor functions, would reduce antibody-dependent enhancement (ADE) [34], as well as reduce the risk exacerbating the hyperinflammatory response often associated with severe COVID-19 development [35], while still benefitting from the natural pathogen clearance mechanism of macrophages.

**Fig 2:**
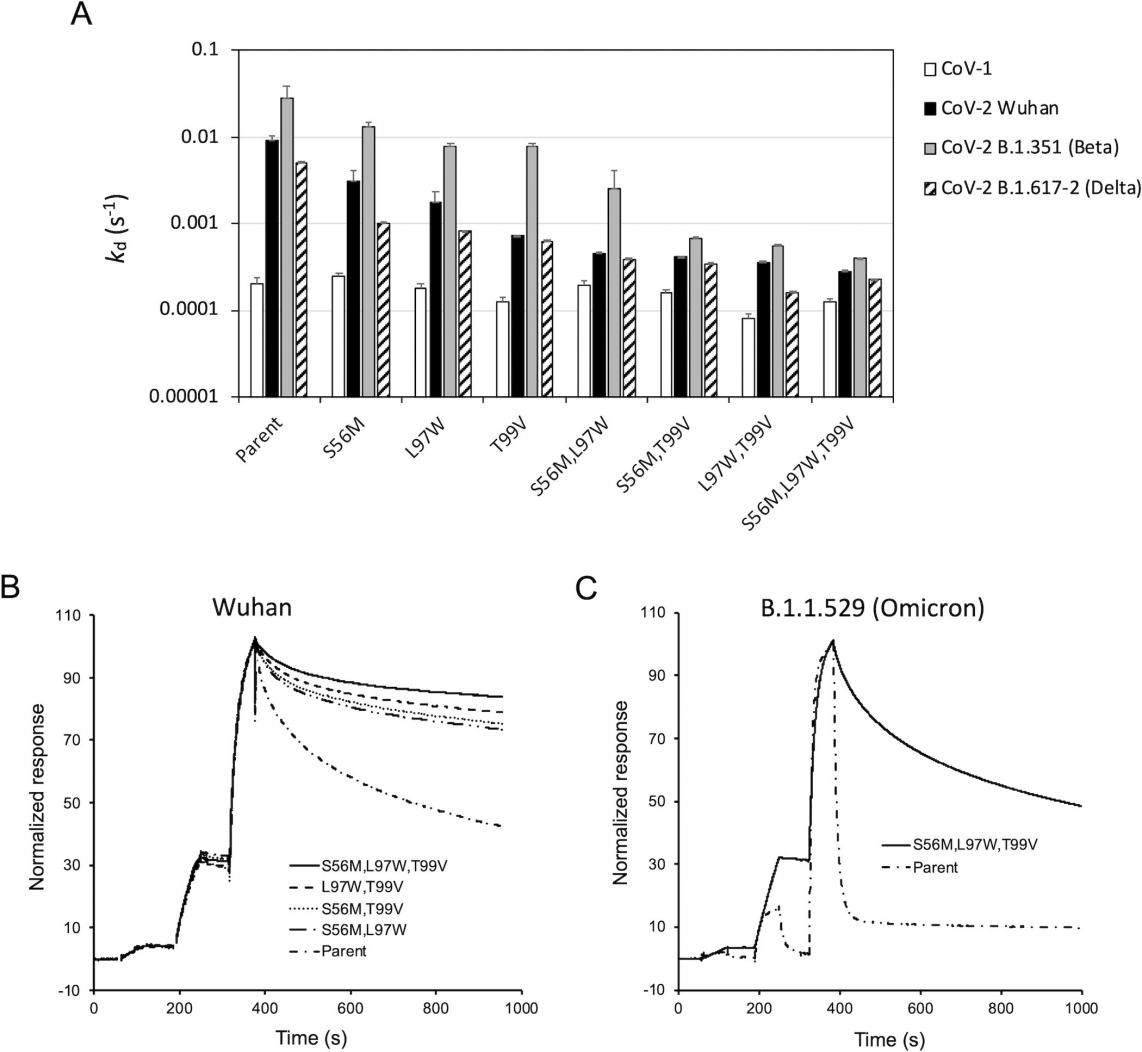
Biophysical testing of VHH-72-Fc mutant hits. (**A**) Dissociation rates (*k*_d_) determined by SPR for the three single-mutant hits and resulting multiple mutants, for binding to immobilized spike RBDs from SARS-CoV-1, SARS-CoV-2 Wuhan, SARS-CoV-2 B.1.351 (Beta) and SARS-CoV-2 B.1.617.2 (Delta). (**B**) Overlaid SPR sensorgrams for the parental variant and the four multiple mutants against immobilized SARS-CoV-2 Wuhan spike RBD. (**C**) Overlaid SPR sensorgrams for the parental variant and the triple mutant against immobilized SARS-CoV-2 B.1.1.529 (Omicron) spike RBD. See also **S3 Fig** for representative sensorgrams and modeled binding fits.

Binding of purified VHH-72-Fc proteins to CoV-1 and CoV-2 S RBDs was carried out by surface plasmon resonance (SPR). Three of the designed single mutants, T99V and L97W from CDR3 and S56M from CDR2, showed improved binding to the CoV-2 spike RBD domain immobilized on the SPR sensorchip, which was primarily due to reduced dissociation rates, *k*_d_ (**Table 2**). The largest effect was seen for T99V, which showed a 10-fold reduced *k*_d_ relative to the parental VHH-72-Fc, while the smallest improvement was seen for the S56M (**Fig 2A**). The same trend of *k*_d_ improvements was obtained for the B.1.351 (Beta) and B.1.617.2 (Delta) CoV-2 spike RBD variants, albeit the magnitude of improvements depended on the RBD variant. Determination of the melting temperatures by DSC for these three single mutants indicated similar thermal stabilities relative to parental VHH-72-Fc (**Table 2**). These 3 single mutants retained the strong binding of the parental VHH-72-Fc to the CoV-1 S RBD, which was even slightly improved in the case of the T99V mutant. This was expected since the FoldX folding stability scores were used to filter out mutations predicted to be significantly detrimental with respect to thermal stability.

**Table 2:** Binding and stability of single-mutation hits and multiple mutants of VHH-72-Fc.

VHH-72-Fc variant	*k*_d_ (10^−4^ s^-1^) from spike-RBD					*T*_m_1 (°C)	*T*_m_2 (°C)
	CoV-1	CoV-2					
		Wuhan	B.1.351 (Beta)	B.1.617.2 (Delta)	B.1.1.529 (Omicron)		
Parent	2.1 ± 0.4	91 ± 10	290 ± 100	51 ± 2	> 10^5^	63.0	83.2
S56M	2.5 ± 0.3	31 ± 9	130 ± 10	10 ± 0.6	> 10^5^	64.0	83.2
L97W	1.8 ± 0.3	18 ± 6	79 ± 7	8.1 ± 0.3	> 10^5^	62.0	83.5
T99V	1.2 ± 0.2	7.2 ± 0.1	77 ± 6	6.2 ± 0.2	> 10^5^	63.5	83.6
S56M,L97W	2.0 ± 0.2	4.4 ± 0.2	25 ± 20	3.9 ± 0.04	7000 ± 1000	62.6	83.5
S56M,T99V	1.6 ± 0.1	4.1 ± 0.1	6.8 ± 0.3	3.5 ± 0.05	252 ± 3	63.9	83.4
L97W,T99V	0.8 ± 0.1	3.5 ± 0.1	5.6 ± 0.2	1.6 ± 0.02	610 ± 50	62.4	83.6
S56M,L97W,T99V	1.3 ± 0.1	2.8 ± 0.1	3.9 ± 0.1	2.3 ± 0.04	70 ± 1	63.5	83.6

### Structural basis of improving mutations

The three mutated positions of VHH-72 interact with the 17-amino-acid contiguous sequence region 368-LYNSASFSTFKCYGVSP-384 of the SARS-CoV-2 spike protein RBD. These positions are far away from mutations present in most currently-circulating SARS-CoV-2 variants of concern (**Fig 1B**), including B.1.351 (Beta) and B.1.617.2 (Delta), suggesting cross-reactivity with these variants. As virus mutations tend to occur more frequently at the interface with the ACE2 host receptor in the so-called receptor binding motif (RBM) region of the S RBD, which is distinct from the VHH-72 binding interface (**Fig 1B**), it is likely that mutating any of these three positions of VHH-72 alone or in combination would also retain binding to most virus variants. However, some virus variants may also contain mutations in other regions of the S RBD, as demonstrated by the recently emerged SARS-CoV-2 variant B.1.1.529 (Omicron). In this variant, 4 of the 15 mutations in the S-RBD region are located outside of the RBM, and three of them (S371L, S373P and S375F) are located precisely in the aforementioned VHH-72 interacting region (**S1 Fig**). Since these mutations introduce larger amino-acid side chains, it is predicted that binding of VHH-72 scaffold to B.1.1.529 (Omicron) S RBD would be negatively impacted by these mutations.

One of the advantages of rational structure-guided affinity maturation is that it helps to understand the structural basis for improvement of binding affinity. The positions of these three single-point mutations of VHH-72 at the binding interface with the spike RBD are shown in **Fig 1C**. The mutation L97W (from the CDR3 loop) introduced new van-der-Waals contacts and weak polar contacts with the backbone of loop C379-S383 and nonpolar contacts with the aliphatic ring of P384. The adjacent mutation T99V (also from the CDR3 loop) introduces a new hydrophobic contact with the hydrophobic part of the K378 side-chain. Finally, the S56M mutation (from the CDR2 loop) introduces new hydrophobic contacts with the side-chains of F374 and F377. However, on the negative side, this hydrophobic mutation is also in close proximity to polar backbone atoms of other residues in the region L367-F374, and eliminates an H-bond anchor to the backbone carbonyl of N370. Overall, it thus appears that all three improving mutations are based on a positive design, where the gain in binding affinity is due to new interactions between the S protein and the mutated antibodies rather than a negative design based on removing detrimental interactions (e.g., clashes) with the parental antibody. Since all three mutations interact with a contiguous linear epitope and two of the mutations are adjacent within the CDR3 loop, we expected a less-than-optimal additivity of single mutation effects upon combining them into multiple-point mutants, as it was in fact observed experimentally (see next section, *vide infra*).

From a computational method development perspective, it is instructive to examine the false-positive prediction, which in this study amounted to 10 out of the 13 tested mutations. False-positive predictions have been observed and analyzed in previous ADAPT affinity maturation campaigns [14, 15], although the present false-positive rate is somewhat higher. A main class of false-positives seem to be related to mutations that increase the size of hydrophobic residues in very confined regions at the binding interface. This class includes the five false-positive mutations of the two Val residues at positions 100 and 110a in CDR3, where even small side-chain increases to Ile or Leu are not tolerated. It is plausible that in these cases, the computational treatment may not appropriately estimate the balance between the affinity gain from new non-polar contacts and the affinity loss due to incurred steric strain. The other five false-positive mutations, located in the CDR2, form a different class, having parental residues that are polar and relatively well-exposed at the surface of the complex. These mispredictions may be related to certain inaccuracies in estimating the balance between the electrostatic interactions, solvation effects and, in the case of mutating G55, entropic changes.

### Biophysical evaluation of multiple mutants

The three lead single-point mutations from round 1 of ADAPT were carried forward to a second round. All possible combinations of these mutations were used to generate three double mutants and one triple mutant. The multiple mutants had excellent developability profiles, which included: (1) production yields by transient transfection at 25-mL scale in CHO cells ranging between 350–500 mg/L with cell viabilities over 91% at day 7 post transfection; (2) single-step purification by Protein-A affinity chromatography affording >99% pure Fc dimers (by analytical UPLC-SEC and SDS-PAGE/Coomassie staining, **S2 Fig**); (3) high thermal stability characterized by VH-domain melting temperature (*T*_m_1) around 63°C, similar to the parental construct (**S4 Fig** and **Table 2**).

Their binding sensorgrams to the original Wuhan SARS-CoV-2 S RBD determined by SPR are shown in **Fig 2B**. All four multiple mutants show improved binding with reduced dissociation rates relative to the parent VHH-72-Fc antibody as well as the component single mutants. The same behavior was obtained again with SARS-CoV-2 S RBDs from the B.1.351 (Beta) and B.1.617.2 (Delta) variants (**Fig 2A** and **Table 2**). The triple mutant S56M,L97W,T99V displayed *k*_d_ values improved by over 30-, 70- and 20-fold for the Wuhan, B.1.351 (Beta) and B.1.617.2 (Delta) SARS-CoV-2 S-RBD variants, respectively, relative to the parent. These dissociation rates, which are in the single-digit 10^−4^ s^-1^ range, approached that measured against the CoV-1 spike RBD which was maintained between the triple mutant and the parent. The double mutant L97W,T99V closely followed the triple mutant with a similar binding strength against the three CoV-2 virus variants, while also affording a 4-fold improved *k*_d_ from the RBD of CoV-1 relative to the parent (**Table 2**). This suggests that the double mutant already harbors most of the improvements seen with the triple mutant, in agreement with the single mutation data showing that the S56M mutation affords the smallest improvement among the three progressed mutations.

While this paper was under peer review, we were able to test binding of the single hits and multiple leads to SARS-CoV-2 B.1.1.529 (Omicron) S RBD. As predicted structurally, parental VHH-72-Fc and single mutants had very low dissociation constants (**Table 2**). Remarkably, the multiple mutants were able to rescue binding, with the best level attained by the triple mutant S56M,L97W,T99V that reached a *k*_d_ similar to that of the parental antibody against the Wuhan S-RBD variant (**Fig 2C** and **Table 2**). Apparently, the negative impact of the aforementioned mutations in the B.1.1.529 (Omicron) S RBD has been in part offset by the combined new interactions established by the mutations of the antibody triple mutant. This further demonstrates the generality of the dual-affinity optimization approach against SARS coronaviruses from different clades for broad targeting of virus variants within a given clade.

### *In vitro* viral neutralization by lead mutants

**Fig 3:**
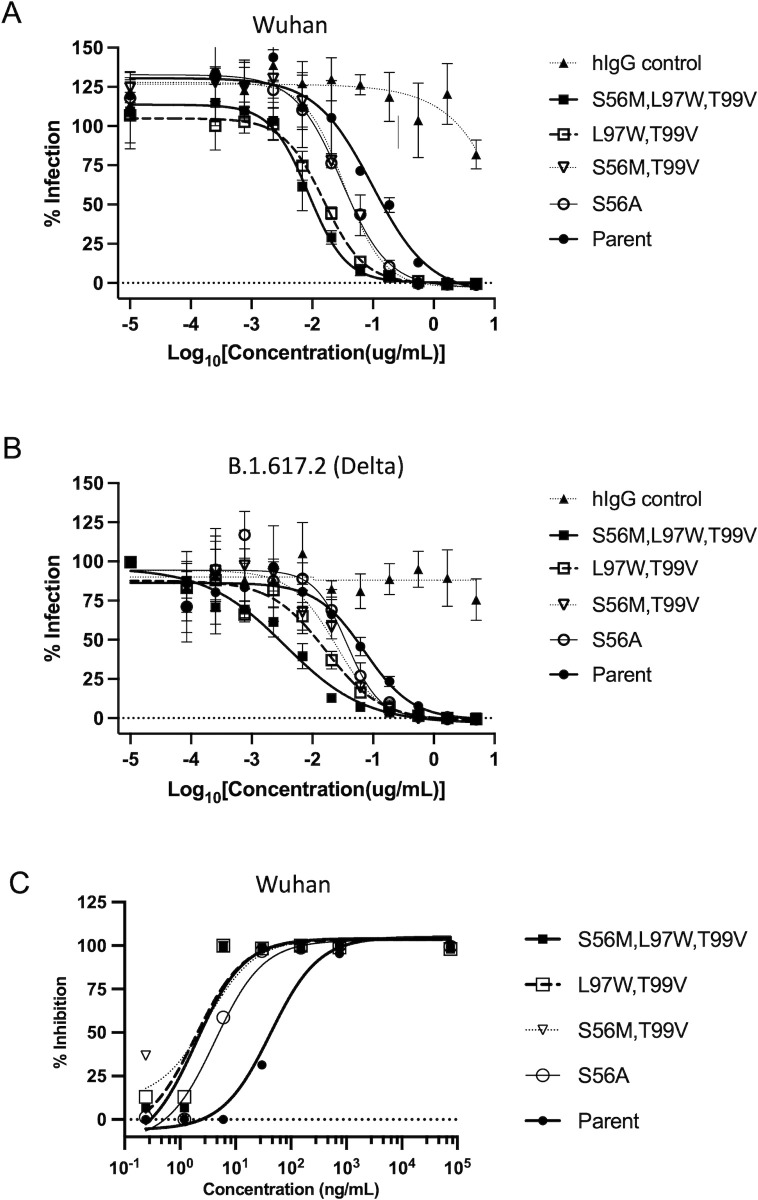
Viral neutralization efficacy by VHH-72-Fc lead mutants. (**A**) Neutralization of VLPs expressing the SARS-CoV-2 Wuhan spike protein for infecting HEK293T cells co-expressing human ACE2 and TMPRSS2. (**B**) Neutralization of VLPs expressing the SARS-CoV-2 B.1.617.2 (Delta) variant spike protein for infecting HEK293T cells co-expressing human ACE2 and TMPRSS2. (**C**) Neutralization of live SARS-CoV-2 Wuhan virus for infecting Vero-E6 cells.

These binding affinity improvements prompted us to test the best three multiple mutants for their ability to block the entry of SARS-CoV-2 pseudotyped virus like particle (VLP) into cells *in vitro*. The first set of experiments employed a non-replicating pseudovirus neutralization assay [30]. In this assay, two different VLPs were produced and tested, containing SARS-CoV-2 spike protein from either the Wuhan or B.1.617.2 (Delta) strains, a luciferase reporter and the minimal set of lentiviral proteins required to assemble the VLPs. A monolayer of HEK293T cells co-expressing human ACE2 and human TMPRSS2 were exposed to the VLPs. The ability to block entry of these particles into cells was detected by loss of signal of the luciferase reporter. As shown in **Fig 3A and 3B** and listed in **Table 3**, the three multiple mutants of VHH-72-Fc blocked cellular infection by the two VLPs more potently than the parental antibody. The best neutralization potencies were obtained with the triple mutant S56M,L97W,T99V, which displayed IC50 values of 9 ng/mL and 4 ng/mL against Wuhan and B.1.617.2 (Delta) spike protein pseudotyped VLPs, respectively, which represent 11-fold and 18-fold improvements, respectively, relative to the parent compound. The double mutant L97W,T99V also showed strong neutralization potencies, amounting to 6-fold and 4-fold improvements, respectively, relative to the parental compound.

**Table 3:** *In vitro* SARS-CoV-2 neutralization efficacy of VHH-72-Fc mutant leads.

VHH-72-Fc variant	IC50 (ng/mL)		
	Pseudovirus		Live Virus Wuhan
	Wuhan	B.1.617.2	
Parent	98	72	43
S56M,T99V	34	25	3
L97W,T99V	16	17	2
S56M,L97W,T99V	9	4	2
S56A	30	37	4

Encouraged by these improvements obtained with the pseudotyped VLP assay, we next employed a microneutralization assay to assess the ability of these three multiple mutants to neutralize infection of human VERO-E6 cells by the live replicating authentic virus. For this test, we used the original CoV-2 Wuhan variant. As shown in **Fig 3C** and listed in **Table 3**, the multiple mutants tested inhibited replication of live virus with IC50 values in the 2–3 ng/mL range, hence affording over 20-fold improved efficacies for the best mutants L97W,T99V and S56M,L97W,T99V relative to the parental VHH-72-Fc. Taken together, these data demonstrate that ADAPT-guided optimization of the VHH-72 paratope for improved CoV-2 spike protein binding affinity can translate into a marked functional improvement at cellular level.

While this work was in progress, another improved mutant of VHH-72 bearing the single point mutation S56A was reported [36]. This mutation was not prioritized by our ADAPT *in silico* scoring protocol, possibly due to the small structural change it introduces (Ala versus Ser). To compare it with our mutants, we introduced this mutation in the Fc fusion context of our mutants. Pseudovirus and live virus neutralization IC50 values are listed in **Table 3** showing the S56A mutation affords only 3-fold and 2-fold improvements in the neutralization of Wuhan and B.1.617.2 (Delta) spike pseudotyped VLPs, respectively, and 11-fold improvement in the neutralization of authentic Wuhan virus, relative to the parental compound. Hence, the variants identified in this work extend the optimization of VHH-72 beyond the levels afforded by the S56A mutation.

### *In vivo* evaluation of viral neutralization efficacy

The lead mutant that performed best in the *in vitro* neutralization assays, S56M,L97W,T99V, was tested *in vivo* for efficacy as a therapeutic agent for neutralizing the SARS-CoV-2 infection. The Syrian hamster model of COVID-19 infection was used [37]. Hamsters were intranasally challenged with 10^4^ PFU of SARS-CoV-2 Wuhan virus and then therapeutically treated 4 hours later with the VHH-72-Fc variant administered as a single intraperitoneal injection of 10 mg/kg. PBS was used as negative control and the parental VHH-72-Fc and its variant carrying the previously reported mutation S56A [36] were used as positive controls. Although prophylactic administration usually leads to more pronounced efficacies, we opted to test these variants in the more relevant scenario appropriate for their eventual intended use as therapeutic agents. Moreover, intraperitoneal administration incurs a further time delay required for drug distribution from the ventral cavity area into systemic circulation to finally reaching the lungs of treated animals.

**Fig 4:**
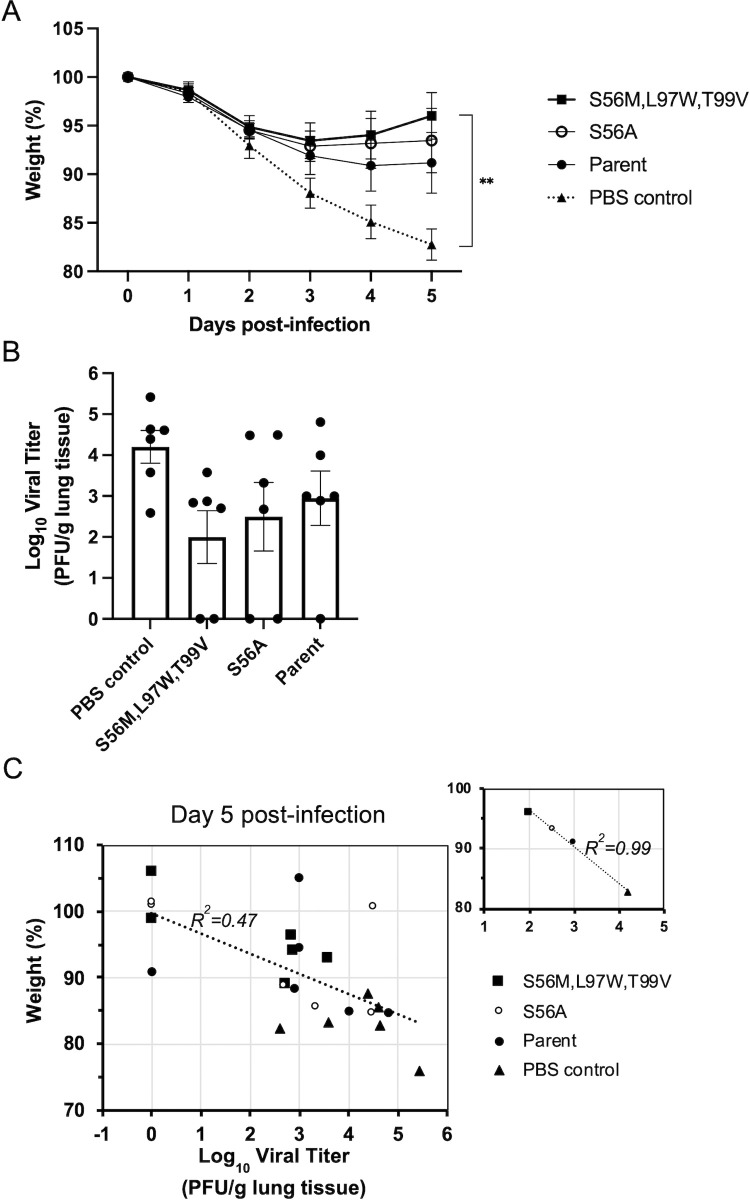
*In vivo* therapeutic efficacy of VHH-72-Fc lead mutant in SARS-CoV-2 infected hamsters. Male hamsters were challenged intranasally with 10^4^ PFU of SARS-CoV-2 Wuhan isolate. Four hours later they were administered test articles intraperitoneally at a dose of 10 mg/kg. Each group consisted of 6 animals. (**A**) Body weight measurements during the time course of the experiment (Mean +/- SEM). (**B**) Live virus titers in lung tissues at day 5 post-infection determined with the plaque assay (Mean +/- SEM). (**C**) Correlation between body weight measured at day 5 post-infection and virus titer in the lungs tissues collected at necropsy (day 5 post-infection) for all animals from all treatment and control groups. The smaller graph on the upper-right plots the correlation between the corresponding mean values in each group.

With this *in vivo* study protocol, the VHH-72-Fc lead optimized mutant S56M,L97W,T99V had a marked impact on the treated group relative to the control group (PBS vehicle alone), as indicated by reduced body weight losses post-infection (**Fig 4A**). The group treated with this triple mutant actually showed body weight gains after day 3 post challenge, which was superior to the recovery afforded by the parental VHH-72-Fc antibody. The group treated with the optimized VHH-72-Fc variant S56M,L97W,T99V also showed a reduction of two orders of magnitude relative to the control group in the average virus titre in lung tissues collected at day 5 post challenge, versus one order of magnitude reduction afforded by the parental VHH-72-Fc (**Fig 4B**). There was a relatively high variability in the virus titre data obtained with the plaque assay for all groups, and hence the observed changes in live virus titres between groups treated with VHH-72-Fc variants did not reach statistical significance. Despite the statistical uncertainty, the average virus titre levels of various groups were correlated with the corresponding average body weight levels at day 5 post infection (**Fig 4C**). Importantly, this *in vivo* efficacy trend was also fully supported by the trends obtained from *in vitro* virus neutralization (**Table 3**). Taken together, these data underscore the therapeutic potential of structure-based optimized VHH-72-Fc for treating COVID-19 infections.

## Conclusions

Structure-based ADAPT-guided affinity maturation helped improve the virus neutralization efficacy of VHH-72-Fc against SARS-CoV-2. This covered the original Wuhan strain as well as some of the other major virus variants of public concern currently in circulation. Simultaneous dual-affinity optimization against sarbetacoronaviruses from distinct phylogenetic clades appears as a sensible strategy for achieving cross-specificity against a majority of virus variants within a given clade. This dual-optimization was feasible mainly due to a relatively conserved S-RBD epitope targeted by VHH-72, which is distinct from that targeted by ACE2 that is more prone to mutational escape from most neutralizing antibodies currently under development. The results reported here should support development of improved biotherapeutics for COVID-19.

## Supporting information

S1 Fig Structure mapping of the mutations occurring in the recently emerged SARS-CoV-2 variant B.1.1.529 (Omicron) relative to VHH-72 and ACE2 binding sites. Rendering is as follows: VHH-72 as green Cα trace, ACE2 receptor ectodomain as black Cα trace and SARS-CoV-2 Wuhan S RBD as gray Cα trace. The 15 amino-acid side chains mutated in the Wuhan S RBD to the variant B.1.1.529 (Omicron) S RBD are rendered as red CPK models. The three mutated residues in the S RBD surface interacting with VHH-72 are indicated by red arrows and labeled.
(TIFF)

S2 Fig Characterization of protein-A-purified lead mutants of VHH-72-Fc. (**A**) Analytical UPLC-SEC chromatograms obtained on a BEH200 column. The peak at ~4.36 min is associated to the buffer and its area was removed from the calculation. (**B**) SDS-PAGE staining (Sypro Ruby staining, load: 1 μg).
(TIFF)

S3 Fig Representative SPR sensorgrams for binding of VHH-72-Fc mutants to immobilized S-RBD variants. Modelled 1:1 fits are shown in red and are overlaid on top of the double-referenced binding data shown in black.
(PDF)

S4 Fig Biophysical testing of VHH-72-Fc mutant leads. Overlaid DSC thermograms for the parental variant and the four multiple mutants.
(TIFF)
