# Supplementary figures and images for: Structure-based dual affinity optimization of a SARS-CoV-1/2 cross-reactive single-domain antibody
Source: PLoS One. 2022 Mar 30;17(3):e0266250. doi: 10.1371/journal.pone.0266250 (PMC8967028; doi:10.1371/journal.pone.0266250)

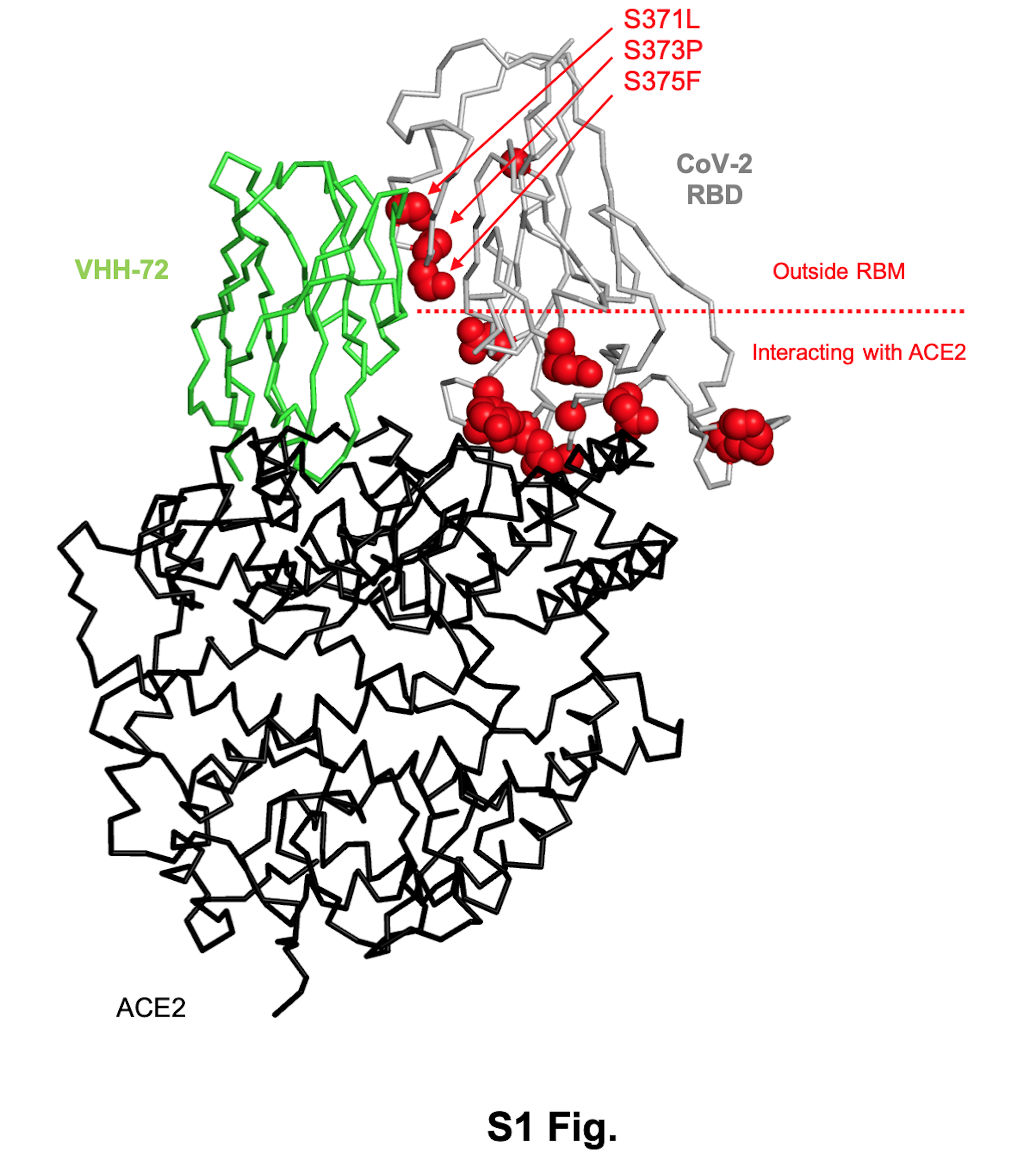

Supplement: S1 Fig — Rendering is as follows: VHH-72 as green Cα trace, ACE2 receptor ectodomain as black Cα trace and SARS-CoV-2 Wuhan S RBD as gray Cα trace. The 15 amino-acid side chains mutated in the Wuhan S RBD to the variant B.1.1.529 (Omicron) S RBD are rendered as red CPK models. The three mutated residues in the S RBD surface interacting with VHH-72 are indicated by red arrows and labeled. (TIFF) [file pone.0266250.s001.tiff]

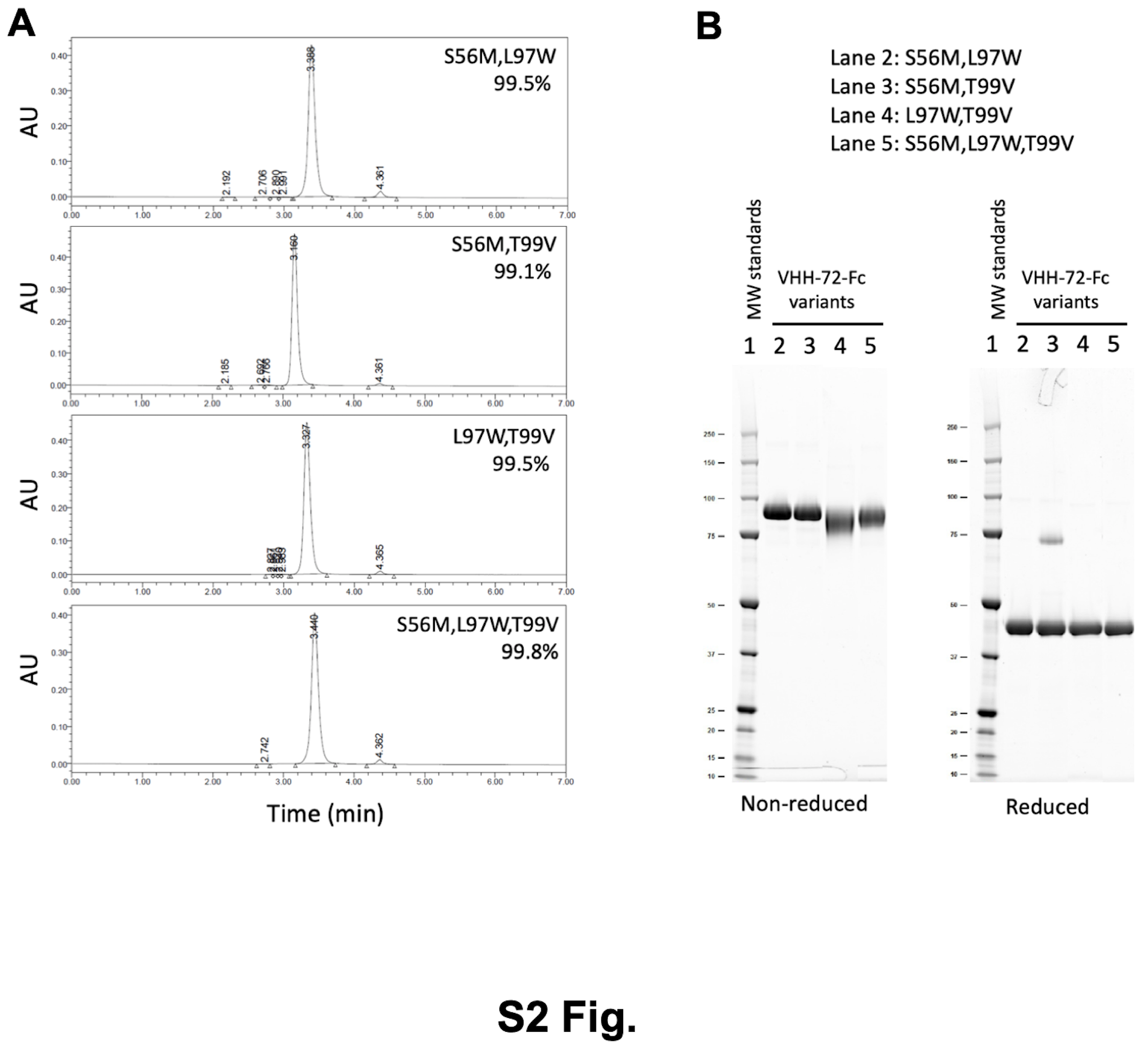

Supplement: S2 Fig — (A) Analytical UPLC-SEC chromatograms obtained on a BEH200 column. The peak at ~4.36 min is associated to the buffer and its area was removed from the calculation. (B) SDS-PAGE staining (Sypro Ruby staining, load: 1 μg). (TIFF) [file pone.0266250.s002.tiff]

CoV-1 S-RBD

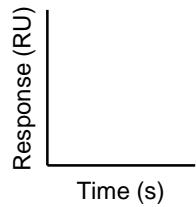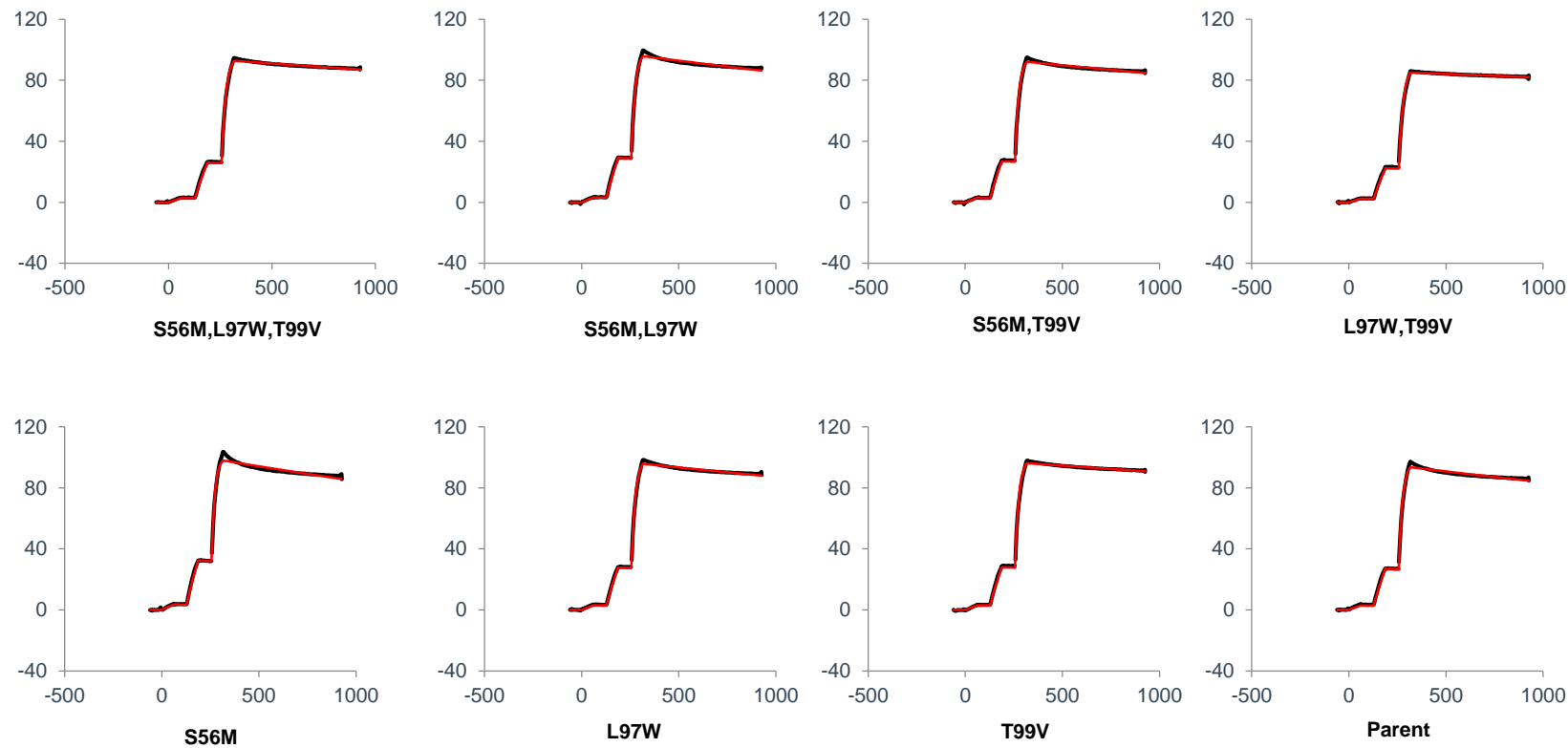

# CoV-2 Wuhan S-RBD

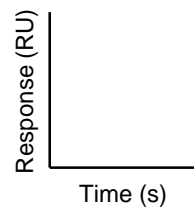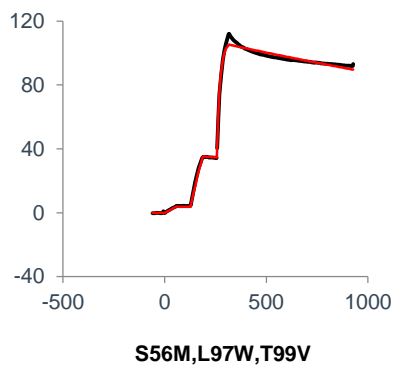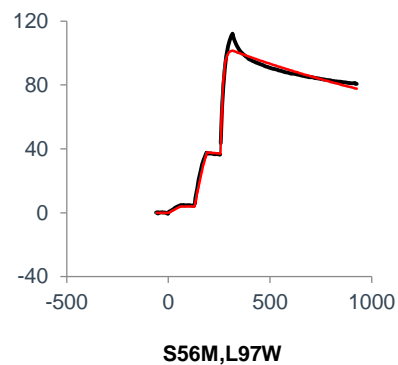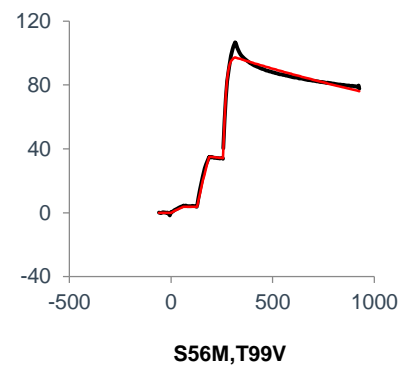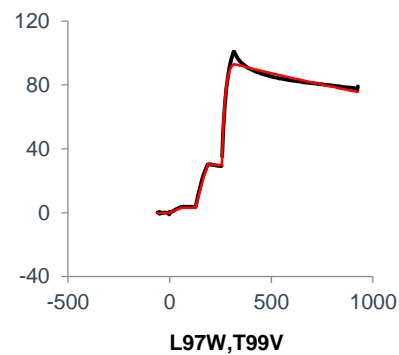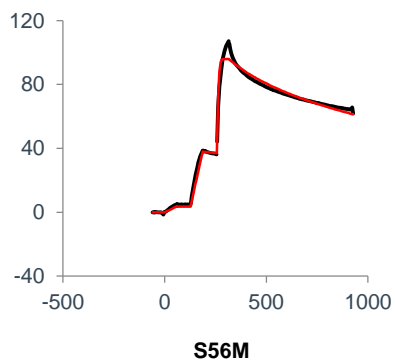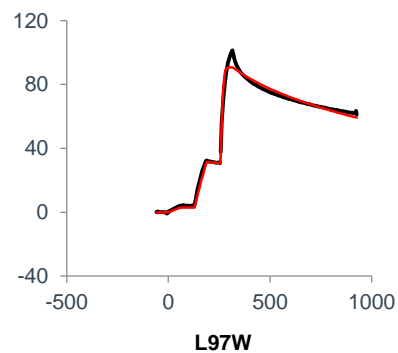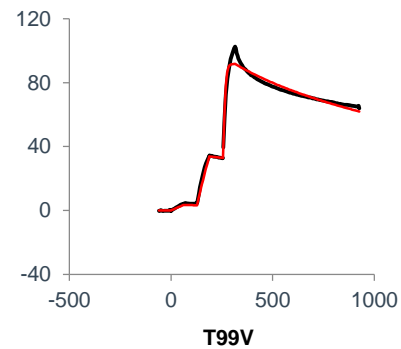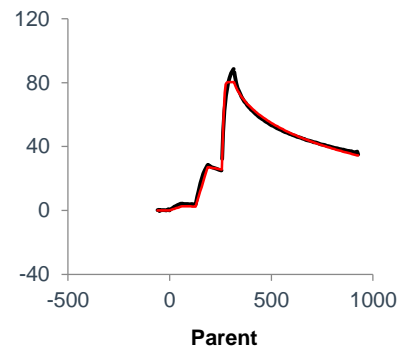

CoV-2 B.1.351 (Beta) S-RBD

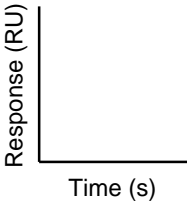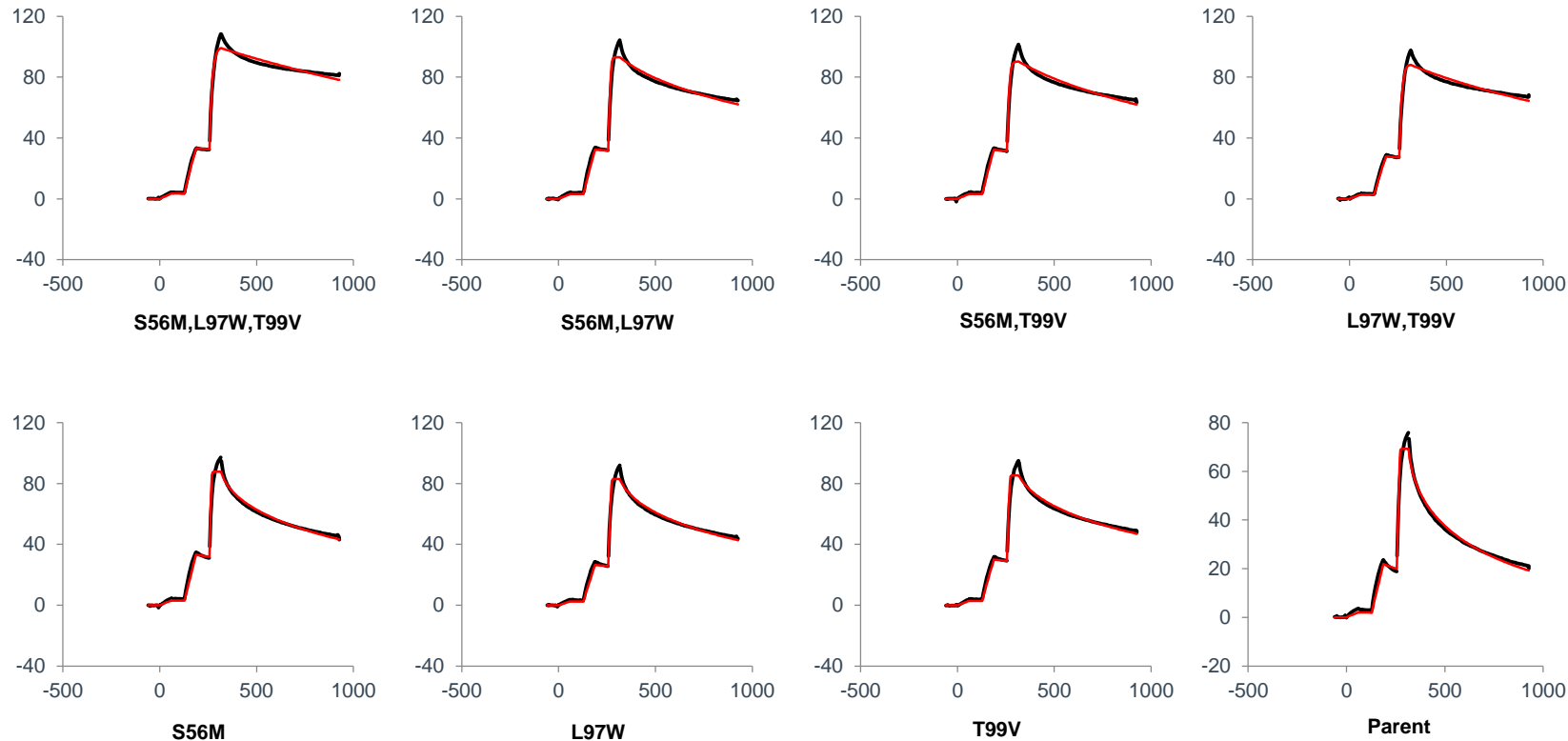

# CoV-2 B.1.617.2 (Delta) S-RBD

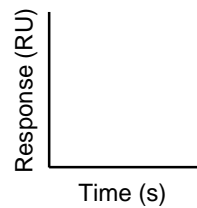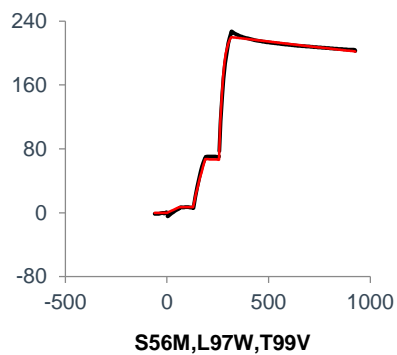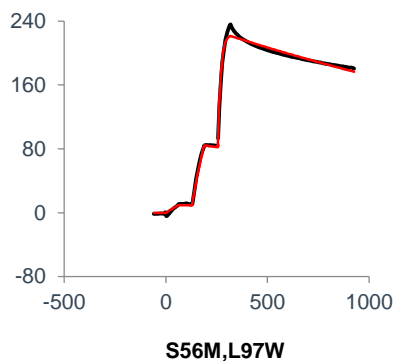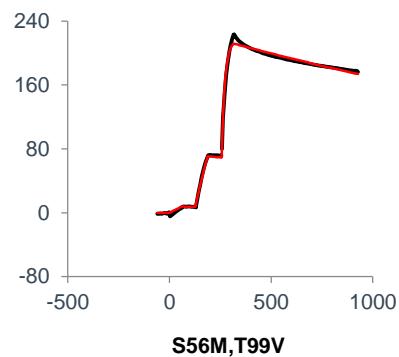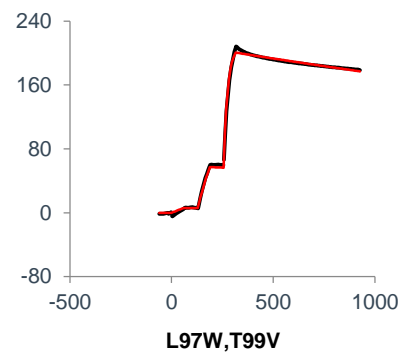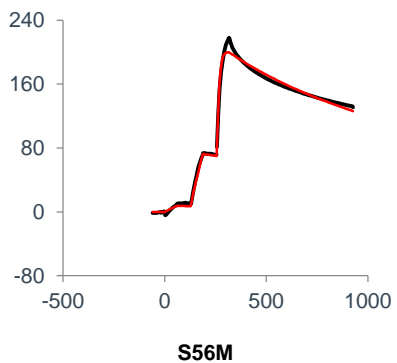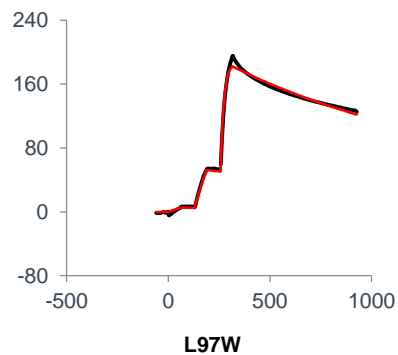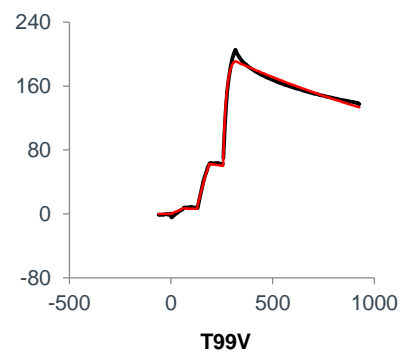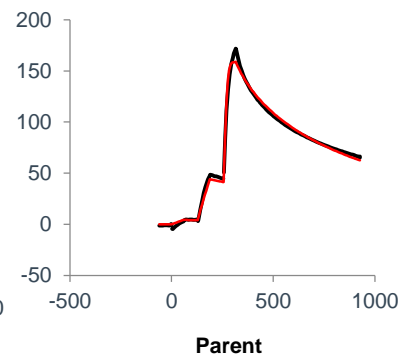

## CoV-2 B.1.1.529 (Omicron) S-RBD

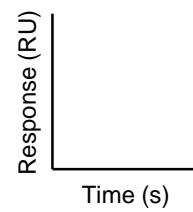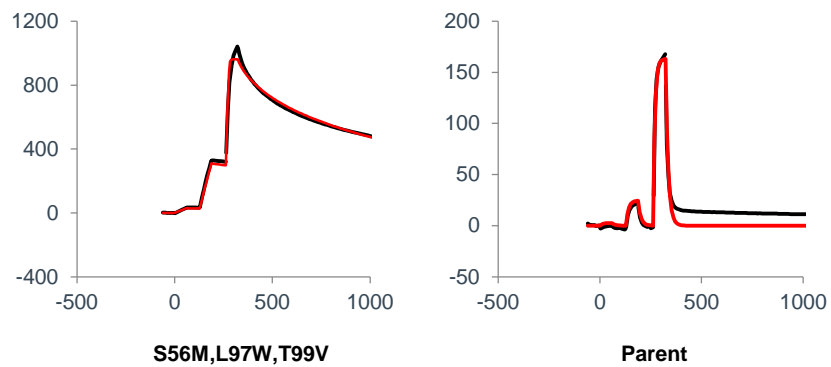

**S3 Fig.**

Supplement: S3 Fig — Modelled 1:1 fits are shown in red and are overlaid on top of the double-referenced binding data shown in black. (PDF) [file pone.0266250.s003.pdf]

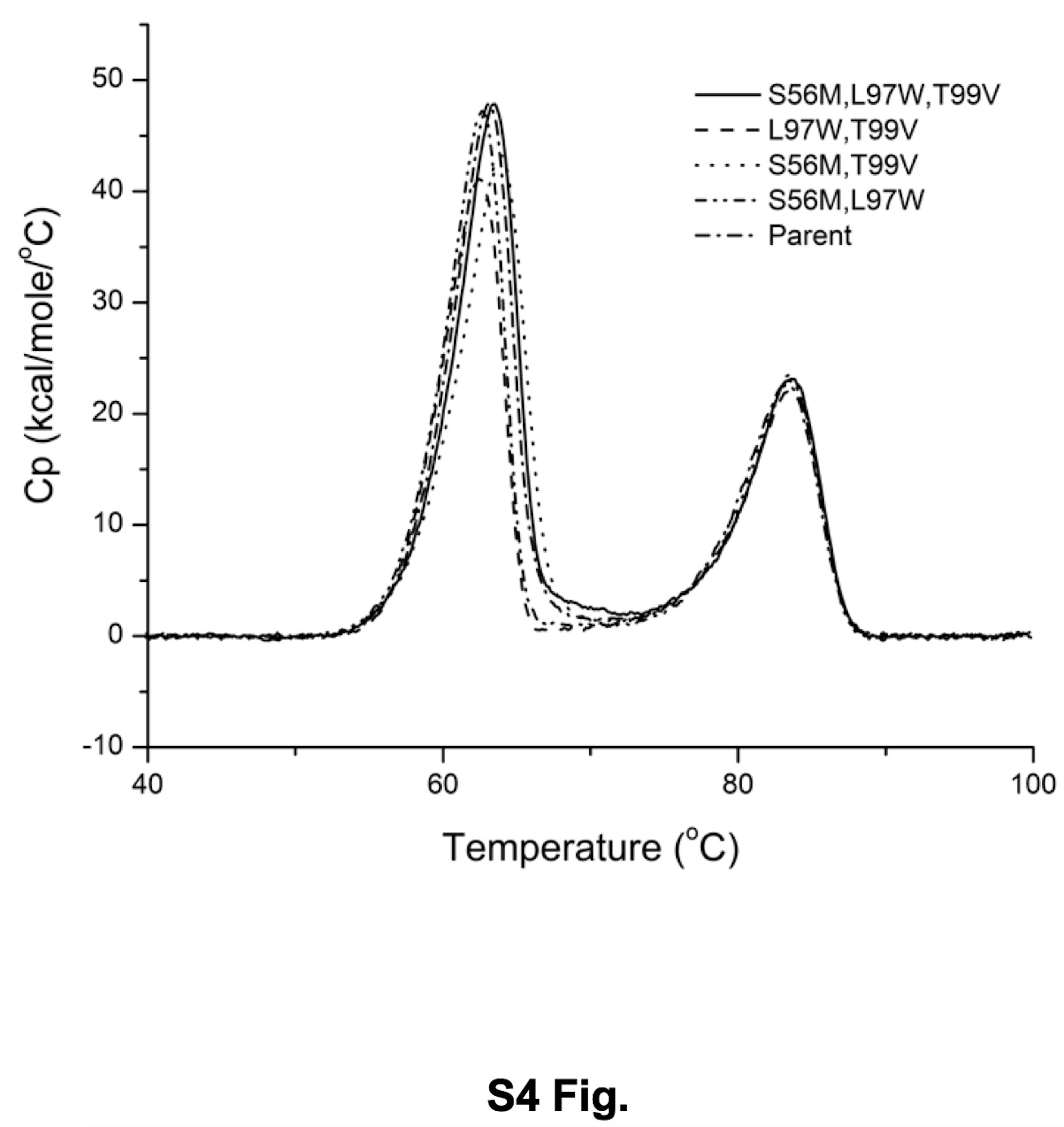

Supplement: S4 Fig — Overlaid DSC thermograms for the parental variant and the four multiple mutants. (TIFF) [file pone.0266250.s004.tiff]
